# Recurrent spontaneous pneumoperitoneum secondary to intestinal dilatation caused by allied disorders of Hirschsprung’s disease: a case report

**DOI:** 10.1186/s12876-022-02376-w

**Published:** 2022-06-27

**Authors:** Yue Yin, Yun Zhang, Bei Tan, Weixun Zhou, Wei Liu, Xuejun Zeng

**Affiliations:** 1 Division of General Internal Medicine, Department of Primary Care & Family Medicine, Peking Union Medical College Hospital, Chinese Academy of Medical Sciences, State Key Laboratory of Complex Severe and Rare Diseases (Peking Union Medical College Hospital), Beijing, 100730 China; 2 Department of Gastroenterology, Peking Union Medical College Hospital (PUMCH), Chinese Academy of Medical Science (CAMS) and Peking Union Medical College (PUMC), Beijing, 100730 China; 3 Department of Pathology, Peking Union Medical College Hospital (PUMCH), Chinese Academy of Medical Science (CAMS) and Peking Union Medical College (PUMC), Beijing, 100730 China; 4 Department of Radiology, Peking Union Medical College Hospital (PUMCH), Chinese Academy of Medical Science (CAMS) and Peking Union Medical College (PUMC), Beijing, 100730 China

**Keywords:** Hirschsprung’s disease allied disorders, Recurrent spontaneous pneumoperitoneum, Gastrointestinal dysmotility, Intestinal dilatation

## Abstract

**Background:**

Allied disorders of Hirschsprung’s disease (ADHD) mainly present with bowel obstruction, intestinal dilatation, and chronic constipation, while recurrent spontaneous pneumoperitoneum was rarely reported. We aimed to report a case of recurrent spontaneous pneumoperitoneum caused by ADHD.

**Case presentation:**

A 59-year-old female patient presented with progressive and severe constipation in the past 30 years. She suffered from abdominal discomfort, which was described as ‘gurgling’ during the last three years. Radiography showed free-air and intestinal dilatation, without any other diseases, and she was identified with recurrent spontaneous pneumoperitoneum. Gastrointestinal transit test indicated gastrointestinal motility disorder, and anorectal manometry confirmed the presence of rectal anus-suppressing reflex. Subtotal colectomy was performed to relieve apparent constipation, and the postoperative pathological examination of the colon demonstrated proliferation of nerve fibers and hyperplasia of myenteric plexuses, as well as a relatively scarcity of ganglion cells in the myenteric plexus. Based on the presentations and the postoperative pathology, she was diagnosed with ADHD. The recurrent spontaneous pneumoperitoneum was regarded as the gas escape from dilated intestines, which was in high pressure. All the symptoms and her mental state were improved after the treatment with gastrointestinal decompression and enteral nutrition. However, during follow-up visits, she had intestinal infection, and suffered from severe diarrhea and water-electrolyte imbalance, and the patient eventually died at 17 months after the diagnosis.

**Conclusion:**

ADHD could be a rare cause of recurrent spontaneous pneumoperitoneum, and are mainly undiagnosed or misdiagnosed. A full-thickness biopsy of the gastrointestinal tract (especially the small intestine and sigmoid colon) and differential diagnosis are recommended for the definitive diagnosis. While the ADHD have shown a poor prognosis, timely and long-term treatment with intestinal decompression and nutritional therapy could help relieve symptoms and provide a better quality of life for such patients.

**Supplementary Information:**

The online version contains supplementary material available at 10.1186/s12876-022-02376-w.

## Background

Allied disorders of Hirschsprung’s disease (ADHD) refer to a disease group that can clinically resemble Hirschsprung’s disease, featuring bowel obstruction, intestinal dilatation, and chronic constipation despite the presence of ganglion cells in the terminal rectum [1]. However, recurrent spontaneous pneumoperitoneum has been rarely reported. In the present study, we reported a case of ADHD with severe and progressive constipation for within 30 years before it was worsened into recurrent spontaneous pneumoperitoneum. Very few such cases have been reported in the English-published articles.

## Case presentation

A 59-year-old woman was admitted to our hospital in June 2019, with chief complaints of constipation for 30 years and recurrent abdominal discomfort for 3 years.

**Fig. 1:**
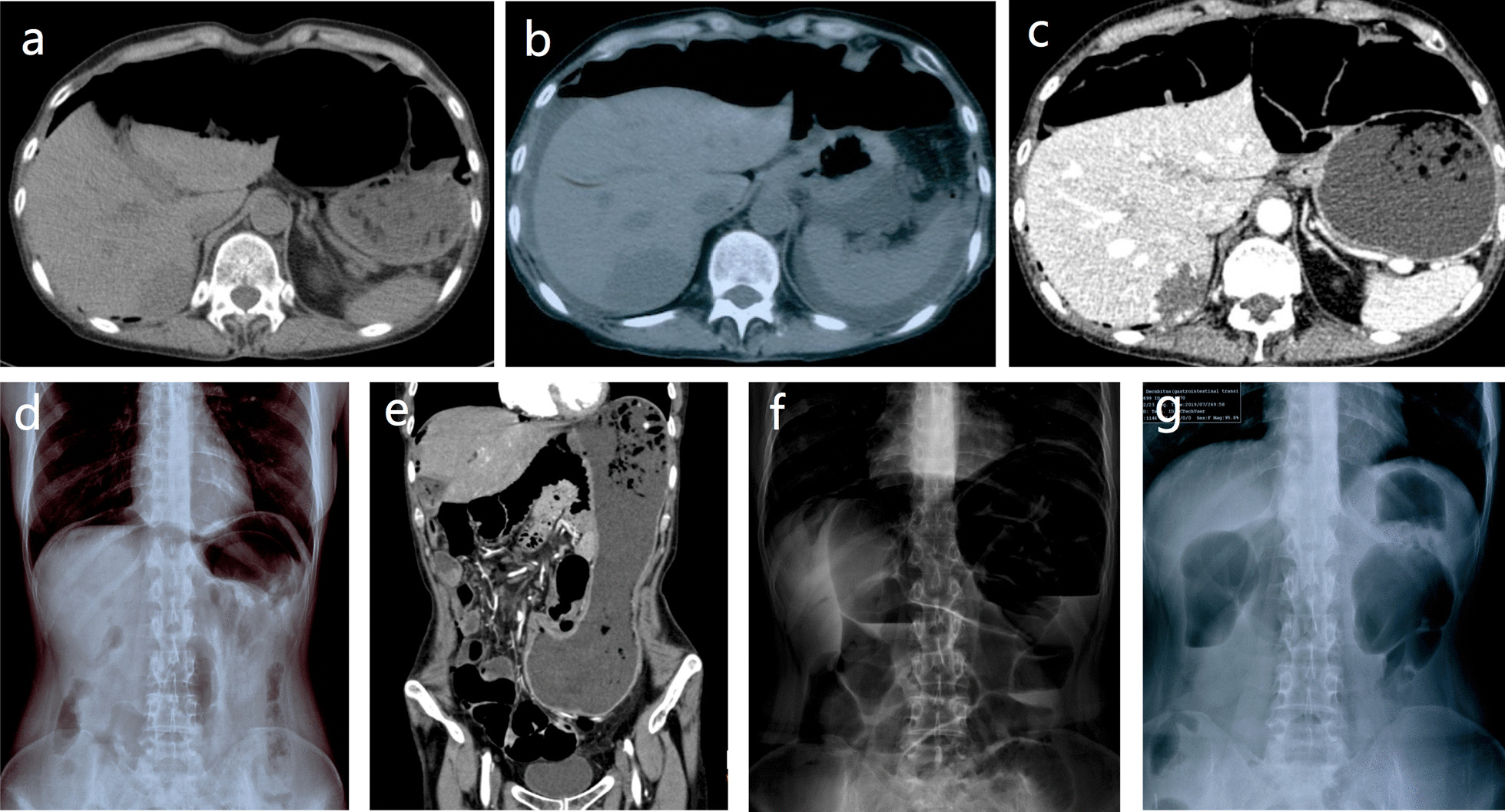
Radiography of abdominal cavity and intestine. **a** Apparently intestinal dilatation and free-air in abdominal cavity in abdominal CT scan. **b** CT scan showing free-air in the peritoneal cavity again with a small amount of perihepatic and perisplenic effusion. **c** Abdominal CT displaying subdiaphragmatic free-air again and intestinal dilatation with recurrence of the symptoms. **d** A translucent crescent or area (free air) below the diaphragm could be observed on upright posteroanterior chest radiography. **e** CT scan revealing marked distension of the stomach, which was vertical and occupied a low position. **f** The upright conventional abdominal radiography showing the presence of intestinal dilatation, air-fluid level formation. **g** Subdiaphragmatic free-air disappeared after gastrointestinal decompression in the upright abdominal radiography

The patient presented with constipation 30 years before her admission. Symptomatic treatments for constipation were given, while symptoms became progressive and severe during these years. In 2016, for no apparent reason, she started to have abdominal discomfort, which was described as ‘gurgling’. She denied having fever or vomiting. She was admitted to a local hospital for further examination. Subdiaphragmatic free-air was shown in the upright conventional abdominal radiography, and free-air in the peritoneal cavity was presented in the computed tomography (CT) scan of the abdomen (Fig. 1a). As gastrointestinal injury was reported as the most common cause of free-air in the peritoneal cavity, laparoscopic exploration was performed immediately. During the surgery, no sign of gastrointestinal perforation was detected, although luminal dilatation of colons was apparent. Then subtotal colectomy was conducted, with no specific findings in the pathological specimens of the colon. Constipation was improved, and the subdiaphragmatic free-air disappeared in X-Ray via gastrointestinal decompression after the surgery. The same ‘gurgling’ recurred in 2017. The CT scan showed free-air in the peritoneal cavity again with a small amount of perihepatic and perisplenic effusion (Fig. 1b), while upper gastrointestinal radiography revealed no contrast agent leakage in the abdominal cavity. Symptomatic treatments, including gastrointestinal decompression, and ultrasound-guided abdominocentesis and catheter drainage were performed. The peritoneal drainage fluid was clear with a total volume of 900 ml, and symptoms were alleviated after treatment. However, in June 2019, similar symptoms and free-air in the abdominal cavity on the CT scan appeared again. She was admitted to our hospital, in order to further investigate the cause of recurrent spontaneous pneumoperitoneum.

Regarding the patient’s past medical history, she had asymptomatic pancytopenia for five years, and she was diagnosed with megaloblastic anemia. Chronic kidney disease (stage 3b) and hepatic hemangioma were also diagnosed. There was no remarkable family history for similar conditions.

Physical examination at the time of admission revealed that the body mass index was 15.6 kg/m^2^. Superficial lymph nodes were not palpable. The examination of lung and heart showed no abnormality. The abdomen was distended, tympanitic, and tender. The liver dullness boundary disappeared, and the shifting dullness was negative. Bowel sounds were active with no abnormality in the rectal examination.

After admission, blood test revealed that pre-existing renal dysfunction was worsened with an increase in creatinine level from 127 to 161 μmol/L (reference range, 45–80 μmol/L) due to insufficient intake. The blood routine test showed no significant differences in data measured before and after admission, and the liver function showed no abnormality. The erythrocyte sedimentation rate and C-reactive protein level were normal. The autoantibody and M protein was negative. Meanwhile, no sign of tumor or inflammation, gastrointestinal perforation, peptic ulcer or pneumatosis cystoides intestinalis was found in the imaging data (Fig. 1c, d). In addition, no obvious abnormality in the gastrointestinal tract mucosa was detected by the endoscopy.

**Fig. 2:**
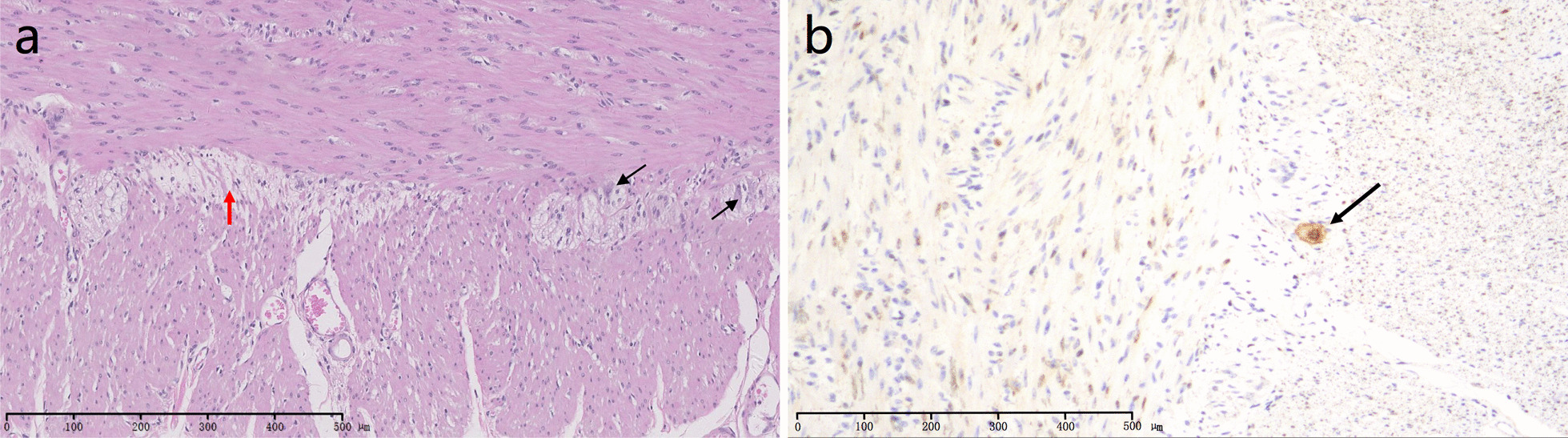
Histological examination of surgical specimens of colon (captured by equipment of Olympus BX51 and acquisition software of Basler Pylon Device). **a** An increased density of myenteric nerve fibers, the presence of giant nerve plexuses, and a relatively scarcity of ganglion cells could be found. The proliferated nerve fibers and hyperplastic myenteric plexuses are indicated with a red arrow. The comparatively few ganglion cells in the myenteric plexus are indicated with black arrows (Hematoxylin and Eosin; original magnification, × 100). **b** NeuN staining shows the ganglion cells (black arrows) in the myenteric plexus

However, the upright conventional abdominal radiography showed the presence of intestinal dilatation, air-fluid level formation, and the absence of mechanical obstruction (Fig. 1e, f). The gastrointestinal transit test showed that the transition of intestinal content during 72 h was 0%, indicating gastrointestinal motility disorder. Anorectal manometry indicated the presence of rectal anus-suppressing reflex, and the sensory threshold of rectus was high. As the immunostaining for neurons was not involved in the pathological examination in 2016, we added it and reviewed the colonic pathological specimens during hospitalization in 2019, which demonstrated proliferation of nerve fibers and hyperplasia of myenteric plexuses, as well as a relatively scarcity of ganglion cells in the myenteric plexus (Fig. 2)

.

Based on the presentations and the postoperative pathology, she was diagnosed with ADHD. According to the multiple-discipline discussion, the recurrent spontaneous pneumoperitoneum was resulted from gas escaped from the dilated intestines under high pressure. The patient was treated with gastrointestinal decompression for several days and received enteral nutrition with the gradually increased intake. Finally, the symptoms of abdominal distention and her mental state were noticeably improved, and subdiaphragmatic free-air disappeared in the upright abdominal radiography (Fig. 1g). The patient’s intake of liquids reached about 2 L/day. Pneumoperitoneum disappeared in imaging findings, the creatinine level was reduced to 121 μmol/L after treatment, and she was discharged in a good clinical condition. During follow-up visits, the patient insisted on enteral nutrition. The abdominal distention was relieved, and she had a relatively stable condition. However, due to the intestinal distention and gastrointestinal motility disorder, the ability of intestinal absorption was poor and she was weak since the disease onset. Regrettably, she had an intestinal infection, leading to severe diarrhea. Due to intestinal malabsorption, the severe dehydration and water-electrolyte imbalance developed, which directly caused the patient’s death at 17 months after the diagnosis. The timeline of disease is shown in Additional file 1.

## Discussion and conclusions

Hirschsprung’s disease is caused by dysperistalsis and a lack of recto-anal reflex due to the aganglionosis of the distal portion of the intestinal tract. Patients with Hirschsprung’s disease generally present in the newborn period with the disordered transit of intestinal content, delayed meconium excretion, abdominal distention, bilious vomiting, or as a young child with severe chronic constipation and intestinal dilatation (megacolon) of the proximal bowel [2].

ADHD were first reported by Ravitch as “pseudo Hirschsprung’s disease” in the Annals of Surgery in 1958 [3], and have been referred to a disease group that clinically resemble Hirschsprung’s disease, despite the presence of ganglion cells in the terminal rectum [1]. According to the literature, ADHD was classified into two categories depending on the pathological findings, which is consisted of seven diseases: (1) abnormal ganglia, including immaturity of ganglia, isolated hypoganglionosis, and intestinal neuronal dysplasia; (2) normal ganglia, including megacystis microcolon intestinal hypoperistalsis syndrome, segmental dilatation of intestine, internal anal sphincter achalasia, and chronic idiopathic intestinal pseudo-obstruction [4].

In this case, the patient had typical symptoms of chronic constipation, serious abdominal distention, and recurrent intestinal obstruction [5]. The imaging findings showed obvious intestinal dilatation and reduction of gastrointestinal motility, which were fully consistent with manifestations of the Hirschsprung’s disease. However, age at disease onset and the presence of rectal anus-suppressing reflex did not support Hirschsprung’s disease. The final diagnosis of ADHD relied on the pathology of colon, indicating hyperplasia of myenteric plexuses and comparatively few ganglion cells in the myenteric plexus, which could be the cause of all her symptoms.

Except for these typical symptoms, this patient presented with spontaneous pneumoperitoneum, which is the rare manifestation of ADHD. Perforation of gastrointestinal tract and laparoscopic surgery are common causes of pneumoperitoneum [6]. Non-surgical causes of pneumoperitoneum are also common, such as mechanical ventilation, spontaneous bacterial peritonitis, scleroderma/collagen vascular disease, pneumatosis cystoides intestinalis, percutaneous endoscopic gastrostomy, chronic peritoneal dialysis, and some gynecologic diseases and miscellaneous causes [6]. In addition, benign spontaneous pneumoperitoneum combined with scleroderma was previously reported, which is related to pneumatosis cystoides intestinalis, and symptoms were improved after conservative treatment [7]. In this case, after thorough screening, the above-mentioned diseases or conditions were not associated with pneumoperitoneum. The spontaneous pneumoperitoneum was recurrent and disappeared after gastrointestinal decompression. As a result, we considered that the spontaneous pneumoperitoneum was related to the high pressure in the intestine. Specifically, degeneration of ganglion cells leads to the intestinal dysmotility, delayed transition of intestinal content, and high pressure, causing gas escape from the dilated intestines and formation of spontaneous pneumoperitoneum.

As for treatment, intestinal decompression and enteral or parenteral nutrition are currently recommended. Radical surgery is controversial and no drug can be recommended to effectively improve functional gastrointestinal disorders or symptoms [4]. In this case, subtotal colectomy was performed because of severe luminal dilatation of colons, and the chronic constipation was relieved, while abdominal distention and intestinal dilatation were still existed and even progressed to recurrent spontaneous pneumoperitoneum. As a result, the positive effect of the surgery on the patient with ADHD could be limited, and full-thickness colonic pathology obtained through surgery provided valuable information to confirm the diagnosis. Thus, for those cases with unclear diagnosis and with severe symptoms of constipation, surgery may be recommended. Importantly, what benefited her was gastrointestinal decompression and enteral nutrition. Regrettably, the patient had intestinal infection that caused severe implications and death, indicating the poor prognosis of the disease.

To our knowledge, this is the first case of ADHD presented with recurrent spontaneous pneumoperitoneum. The patient underwent an surgery and visited doctors from nationwide for the cause of recurrent spontaneous pneumoperitoneum. She was finally diagnosed with ADHD and her symptoms were improved through conservative treatment. ADHD is rare and mainly undiagnosed or misdiagnosed. Physicians are advised to consider ADHD in these patients after differential diagnosis. A full-thickness biopsy of the gastrointestinal tract (especially the small intestine and sigmoid colon) is recommended for the definitive diagnosis [4]. While the disease has a poor prognosis, timely and long-term treatment with intestinal decompression and nutritional therapy could help relieve symptoms and provide a better quality of life for such patients.

## Supplementary Information

**Additional file 1**. The timeline of disease in this case.

## Data Availability

All data generated or analyzed during this study are included in this published article.

## Abbreviations

ADHD: Allied disorders of Hirschsprung’s disease
CT: Computed tomography

## References

[1] Holschneider A, Puri P (2008). Hirschsprung’s disease and allied disorders.

[2] Taguchi T, Ieiri S, Miyoshi K (2017). The incidence and outcome of allied disorders of Hirschsprung's disease in Japan: results from a nationwide survey. Asian J Surg.

[3] Ravitch MM (1958). Pseudo Hirschsprung's disease. Ann Surg.

[4] Muto M, Matsufuji H, Taguchi T (2018). Japanese clinical practice guidelines for allied disorders of Hirschsprung's disease, 2017. Pediatr Int.

[5] Qiu J, Yang G, Lin A (2019). Allied disorders of Hirschsprung's disease. Tech Coloproctol.

[6] Tanner TN, Hall BR, Oran J (2018). Pneumoperitoneum. Surg Clin N Am.

[7] Balbir-Gurman A, Brook OR, Chermesh I, Braun-Moscovici Y (2012). Pneumatosis cystoides intestinalis in scleroderma-related conditions. Intern Med J.

