# Supplementary material for: Recurrent spontaneous pneumoperitoneum secondary to intestinal dilatation caused by allied disorders of Hirschsprung’s disease: a case report
Source: BMC Gastroenterol. 2022 Jun 27;22:316. doi: 10.1186/s12876-022-02376-w (PMC9235130; doi:10.1186/s12876-022-02376-w)
Supplement: Supplementary file 1 — Additional file 1. The timeline of disease in this case. [file 12876_2022_2376_MOESM1_ESM.docx]

**Additional file 1**

| **Age/Time** | **Symptom** | **Examination** | **Treatment** | **Outcome** |
| --- | --- | --- | --- | --- |
| **In her 20s** | Recurrent constipation | None | Laxative | Constipation was gradually worsened. |
| **At the age of 56 years old (in 2006 at a local hospital)** | Abdominal discomfort described as ‘gurgling’ | Subdiaphragmatic free-air on X-ray and free-air in the peritoneal cavity on the CT scan;  Luminal dilatation of colons and no perforation on laparoscopic examination | Subtotal colectomy | Symptoms improved;  Subdiaphragmatic free-air disappeared on X-Ray. |
| **At the age of 57 years old (in 2007 at a local hospital)** | Recurrent abdominal distention | Free air in the peritoneal cavity and perihepatic and perisplenic effusion on the CT scan; no sign of gastrointestinal perforation | Gastrointestinal decompression;  Abdominocentesis anddrainage of the effusion | Abdominal distension and discomfort got improved immediately, while symptoms remained recurrent. |
| **At the age of 59 years old (in 2009 at our hospital)** | Apparent abdominal distention and discomfort after eating a lot | X-ray: intestinal obstruction;  Anorectal manometry: normal rectal anus suppresses reflex anorectal manometry;  Pathology: hyperplasia of myenteric plexuses and comparatively few ganglion cells in the myenteric plexus;  Diagnosis: confirmation of ADHD | Gastrointestinal decompression and enteral nutrition;  Education on lifestyle | Symptoms were improved for one year;  Intestinal infection later on;  The patient died of severe diarrhea and water-electrolyte imbalance at 17 months after the diagnosis. |
